# Cell cycle synchronisation of *Trypanosoma brucei* by centrifugal counter-flow elutriation reveals the timing of nuclear and kinetoplast DNA replication

**DOI:** 10.1038/s41598-017-17779-z

**Published:** 2017-12-14

**Authors:** Corinna Benz, Frank Dondelinger, Paul G. McKean, Michael D. Urbaniak

**Affiliations:** 1 Biomedical and Life Sciences, Faculty of Health and Medicine, Lancaster University, Lancaster, LA1 4YG UK; 2 Lancaster Medical School, Faculty of Health and Medicine, Lancaster University, Lancaster, LA1 4YG UK

## Abstract

We report an optimised centrifugal counter-flow elutriation protocol for the rapid and direct isolation of G1 cell cycle synchronised populations of both the procyclic and bloodstream form stages of *Trypanosoma brucei* that yields viable and proliferative cells. The high quality of the synchronisation achieved can be judged by the uniform DNA content, narrow size distribution, synchronous division, and the maintenance of synchronicity into subsequent cell cycles. We show that early-eluting fractions represent different G1 subpopulations that progress through the cell cycle with distinct temporal profiles post-elutriation, as exemplified by the observation of the maturation of a second flagellar basal body in late G1 phase, DNA replication in S phase, and dimethylation of histone H3 in mitosis/cytokinesis. We use our temporal observations to construct a revised model of the relative timing and duration of the nuclear and kinetoplast cell cycle that differs from the current model.

## Introduction

The eukaryotic cell division cycle is a tightly controlled process that is evolutionarily conserved and of fundamental importance in cell biology. The temporal control of proteins involved in the regulation and progression of cell-cycle is essential to ensure correct growth and division, and is achieved by regulation at multiple levels. A reliable method for cell cycle synchronisation is an invaluable tool to study cell cycle regulation in any organism or cell type. In addition, a non-invasive technique with minimal adverse effects on cell proliferation is desirable to avoid experimental artefacts.

The kinetoplastids, a divergent group of unicellular eukaryotes including the human and animal pathogen *Trypanosoma brucei*, display atypical genome organisation and regulation of gene expression. Protein coding genes are transcribed in polycistronic transcription units of functionally unrelated genes which are co-transcriptionally processed by regulated 5′ *trans* splicing and 3′ polyadenylation to mature mRNA. Further regulation of gene expression can occur through differential export from the nucleus, access to polysomes^1^, and RNA stability^2^. Regulation is thought to be modulated by RNA binding proteins (RBPs)^3^ and there is growing evidence of the importance of RBPs in controlling lifecycle specific gene expression^4–6^. This unusual biology makes *T. brucei* an excellent model system to study post-transcriptional mechanisms of gene regulation.

The cell cycle of *T. brucei* is highly organised and tightly controlled, reflecting the need to co-ordinate not only nuclear division, but also the division and segregation of the mitochondrial kinetoplast DNA and its single copy organelles such as the ER, Golgi and flagellum^7–9^. The timing of nuclear (N) and kinetoplast (K) DNA division differs, thus cells progress from first 1N1K to 1N2K and persist as 2N2K for a defined period prior to cytokinesis, providing a convenient method to characterising their cell cycle positon by DNA content. Although many cell cycle regulators are conserved in trypanosomes, some are missing, and many trypanosome-specific regulators have been identified. Despite the paucity of transcription factor - mediated regulation of gene expression, *T. brucei* regulates its transcript abundance over the cell cycle^10^.

Whilst our knowledge of cell cycle regulation in *T. brucei* has greatly increased over the last decade^7,8,11,12^, a comprehensive picture of cell cycle complexity and interplay of all molecules involved has yet to emerge. The use of live–cell imaging techniques to follow the progress of individual cells across the cell cycle is challenging due to the rapid motility of the parasites. Instead, *in silico* approaches have been used to assign electron- or fluorescence- microscope images of fixed asynchronous cells to defined points of the cell cycle^13–17^, but the approach is technically demanding and time consuming. System-wide approaches such as proteomics that are capable of capturing post-translational modifications have been hampered by the absence of a reliable and reproducible cell cycle synchronisation method that is feasible with the large numbers of cells required, especially for the bloodstream form (Bsf) life stage.

Methods that have been successfully used for synchronisation of *T. brucei* include whole cell culture synchronisation protocols such as starvation and recovery^18^ or hydroxyurea-mediated S-phase arrest and release^19^, and separation techniques such as flow cytometry cell-sorting^13,20^ and centrifugal counter-flow elutriation^10^. The disadvantages of the whole cell culture synchronisation protocols are the potential for artefacts caused by the stress of nutrient deprivation or chemical inhibition and questionable validity of the synchronisation achieved^21,22^. Flow cytometry cell-sorting requires addition of a vital DNA dye to allow cells to be sorted based on their DNA content, but sorting is rather slow (~1 × 10^6^ cells/h). It cannot separate early- from late- G1 stage cells, and whilst the sorted cells remain viable, the majority do not proliferate^20^. Centrifugal counter-flow elutriation is a non-invasive technique that separates particles hydrodynamically in a special centrifugation chamber^23^. At a constant centrifugal speed, an incremental increase in flow rate of elutriation buffer through the chamber is used to wash cells out of the chamber in a size-dependent manner. Elutriation has previously been used to synchronise Pcf cells using a ‘double-cut’ method that requires two sequential elutriation runs, but synchronisation of Bsf stage was not achieved^10^.

Here, we report an optimised protocol for the rapid and direct isolation of tightly G1-synchronised Bsf and Pcf *T. brucei* cell populations by counter-flow centrifugal elutriation. We isolate sub-populations of G1 phase cells that are indistinguishable by flow cytometry but progress synchronously through the cell cycle with distinct temporal profiles post-elutriation. We use our temporal data to model the duration of the cell-cycle periods and the relative timing of the kinetoplast and nuclear cycles.

## Results

### Elutriation of procyclic form cells

**Figure 1:**
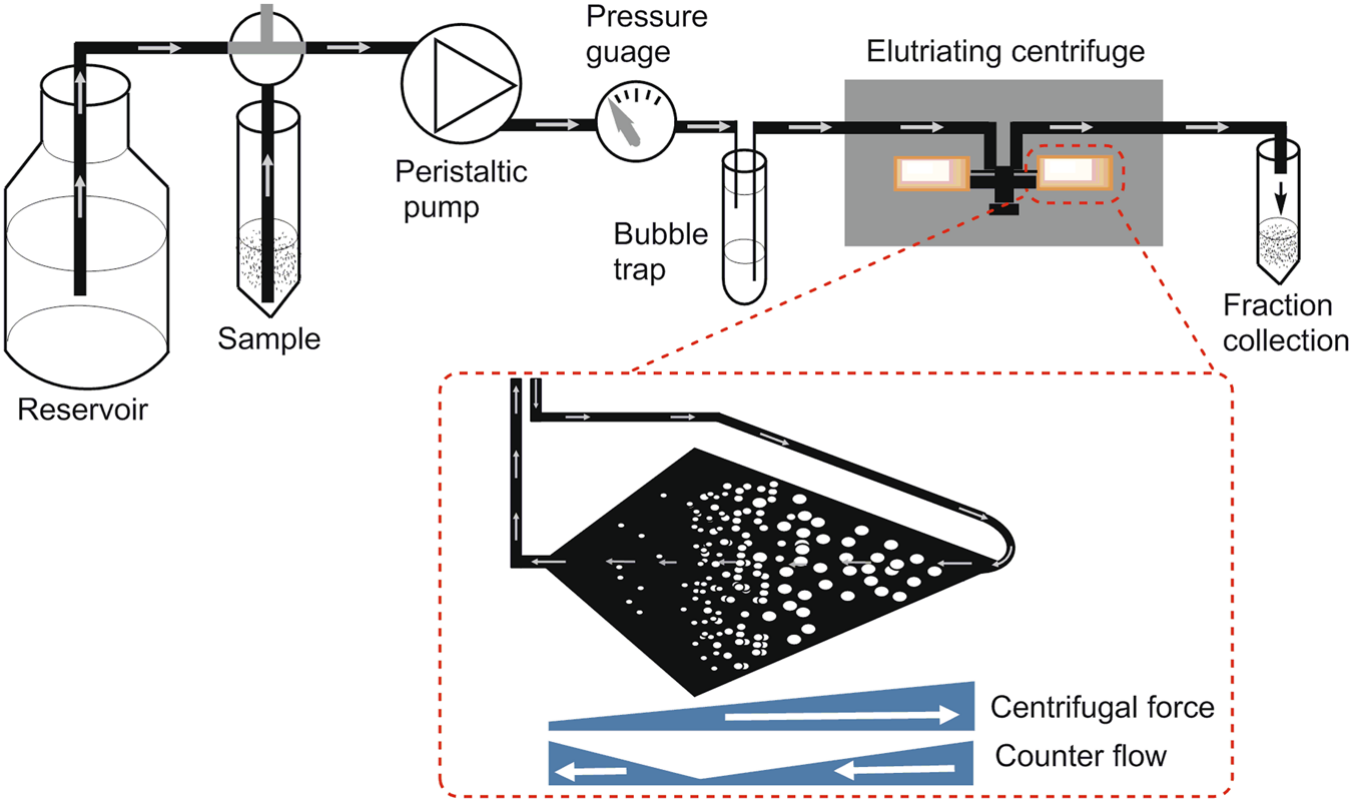
Counter flow centrifugal elutriation of *Trypanosoma brucei*. The elutriation chamber is centrifuged at a constant speed with continuous flow of buffer. Cells are introduced and retained in the chamber at low flow rate, and sequential increases in the flow rate forces the cells to elute from the chamber in size order, with smallest cells eluting first.

In order to produce tightly synchronised *T. brucei* cell populations in sufficient quantities for downstream analysis, we investigated the use of counter flow centrifugal elutriation (Fig. 1). To judge the efficiency of separation we loaded Pcf cells into the elutriation chamber at a low flow rate then incrementally increased the flow rate to elute fractions containing cells of increasing size. Analysis of the cell cycle stages of these fractions by flow cytometry showed that early eluting fractions (18, 20 and 22 ml/min) contained highly enriched G1 populations (>80%), whereas later fractions were more mixed (Supplementary Fig. S1). The first fraction (15 ml/min) yielded low numbers of cells and was frequently contaminated with cell debris, and so was discarded. Whilst only a small proportion of the input material was recovered per fraction (2–10%), efficient separation of G1 populations could be achieved with up to 3 × 10^9^ cells in less than an hour. The flow cytometry analysis was corroborated by analysis of DAPI-stained slides to determine the number of nuclei and kinetoplasts present in each cell (Supplementary Fig. S2). Here, minor differences in the early eluting G1 fractions (18, 20 and 22 ml/min) became apparent with the percentage of cells with one nucleus and a dividing kinetoplast (1N1Kd) in the three fractions increasing steadily with the increase in flow rate used to elute the cells (from 3.2% to 15.8% 1N1Kd).

**Figure 2:**
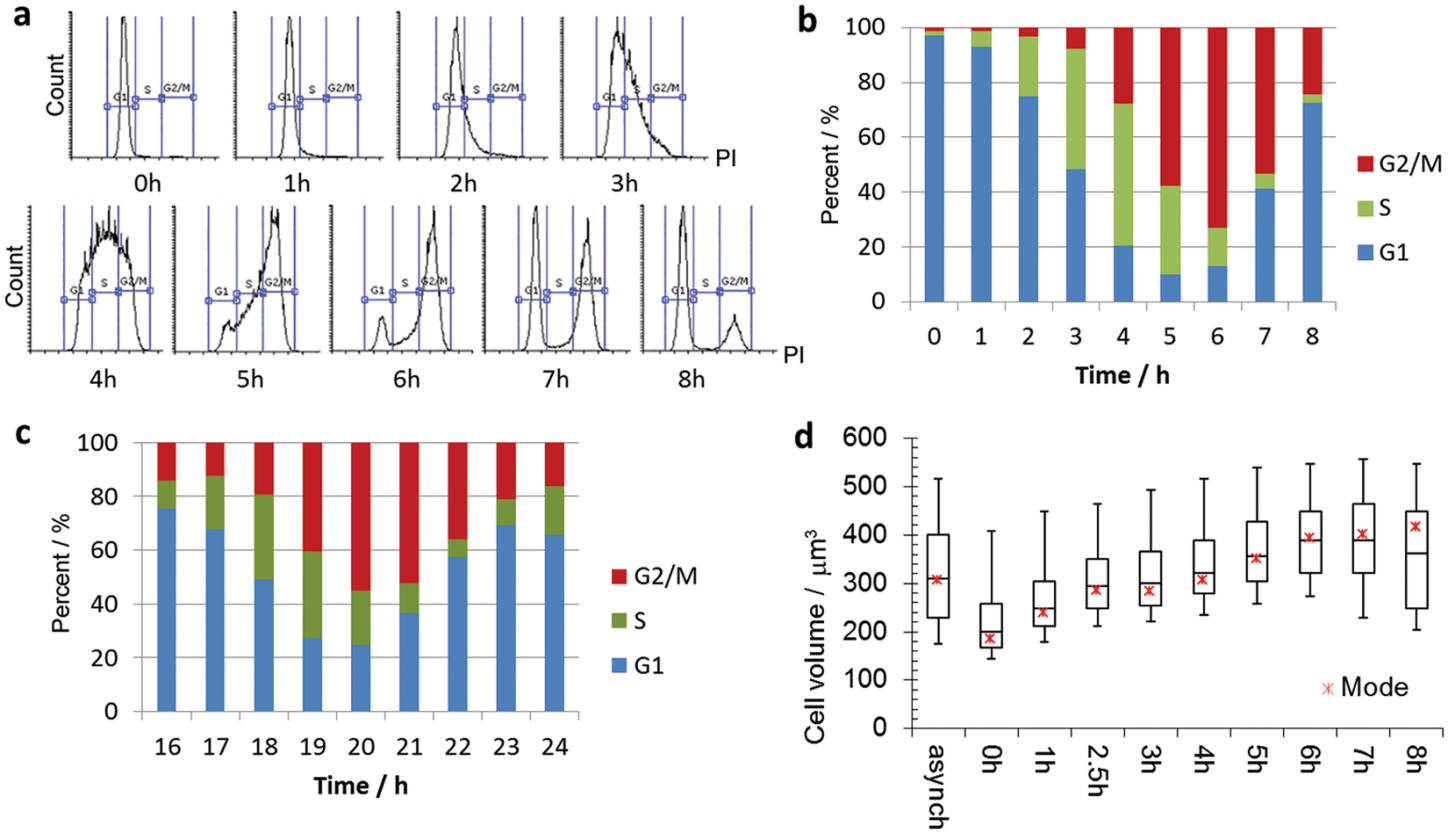
Time-course of early-G1 synchronised Pcf *T. brucei*. Cells samples were withdrawn hourly, stained with PI and analysed by flow cytometry (n = 50,000). (**a**) Flow cytometry trace of first cell cycle post-elutriation representing the PI signal (horizontal axis) versus cell count (vertical axis). (**b**) Bar chart of flow cytometry data for the first cell cycle post elutriation. (**c**) Bar chart of flow cytometry data for the third cell cycle post-elutriation. (**d**) Box plot of cell dimensions determined by CASY model TT (n > 1,500). Boxes represent the median and central quartiles, whiskers represent 9 and 91% of data, and * the mode.

When parasites isolated in the earliest-eluting G1 fraction (18 ml/min, 97% G1) were placed back into culture immediately post-elutriation, they proliferated synchronously without any appreciable lag (Fig. 2, Supplementary Fig. S3). Analysis of samples collected at hourly intervals by flow cytometry revealed that the maximum number of cells in S phase (51%) was reached after 4 hours, and the maximum of number of cells in G2/M phase cells (73%) was reached after 6 h (Fig. 2b), representing an enrichment over asynchronous cells of 3.4-fold and 3.2-fold respectively. The cell division time for logarithmically dividing Pcf cells in culture is 8.5 hours, and after 8 hours 73% of cells had re-entered G1 phase with 24% still in G2/M. Remarkably, cell-cycle synchronisation was still discernible in the third cell cycle following elutriation, reaching 32% S phase after 18 and 19 hours, and 55% G2/M phase after 20 hours (Fig. 2c).

The average cell size for the initial G1 fraction was significantly smaller (p-value 3 × 10^–269^) and had a narrower distribution than the original asynchronous population, confirming that size selection had occurred (Fig. 2d, Supplemental Table S1). The average cell size increased as cells progressed through the cell cycle without an increase in cell count, with the narrow distribution maintained until cells progressed through cytokinesis (Supplementary Fig. S1), where the distribution broadened reflecting the bimodal size distribution of cells in G1 and G2/M.

### Early eluting Pcf fractions represent early, mid and late G1 phase cells

**Figure 3:**
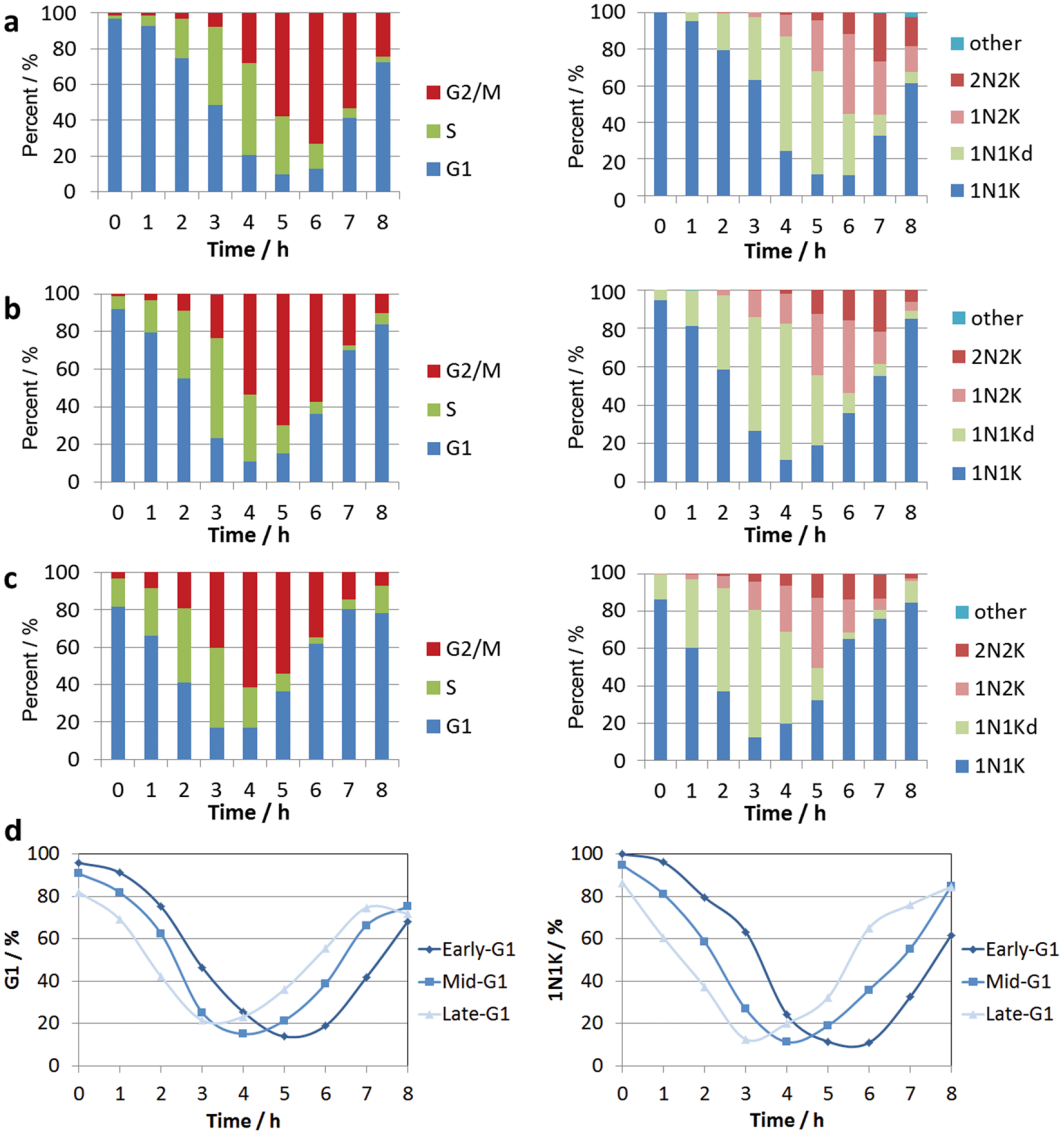
Flow cytometry time-course of distinct G1 synchronised Pcf *T. brucei*. (**a**) Early-G1 cells (97% G1) eluted at 18 ml/min, (**b**) Mid-G1 cells (92% G1) eluted at 20 ml/min, (**c**) Late-G1 cells (82% G1) eluted at 22 ml/min. (**d**) Direct comparison of the proportion of G1 (left) and 1N1K cells (right) over time for the Early-, Mid- and Late-G1 fractions. Left panel - Flow cytometry of PI stained cells at hourly intervals post elutriation (n = 50,000 per time point). Right panel – Microscopy of DAPI stained cells (n > 200 per time point).

When parasites isolated at 20 ml/min (92% G1) and 22 ml/min (82% G1) were placed back into culture immediately post-elutriation and analysed at hourly intervals by flow cytometry, it was apparent that the different fractions progressed through the cell cycle with similar kinetics but with distinct starting points (Fig. 3, Supplemental Fig S4). The observed differences were consistent with the three size-separated fractions representing distinct G1-sub populations of early-, mid- and late-G1 cells. As flow cytometry is unable to distinguish the kinetoplast synthesis and duplication due to its small size in comparison to the nucleus, samples were stained with DAPI and the number of nuclei and kinetoplast per cell analysed by microscopy (Fig. 3), confirming that the fractions were indeed distinct. Notably, the observation of a high proportion of cells with a dividing kinetoplast (1N1Kd) by DAPI microscopy was temporally correlated with the observation of a high proportion of cells judged to be in nuclear S phase by flow cytometry.

**Figure 4:**
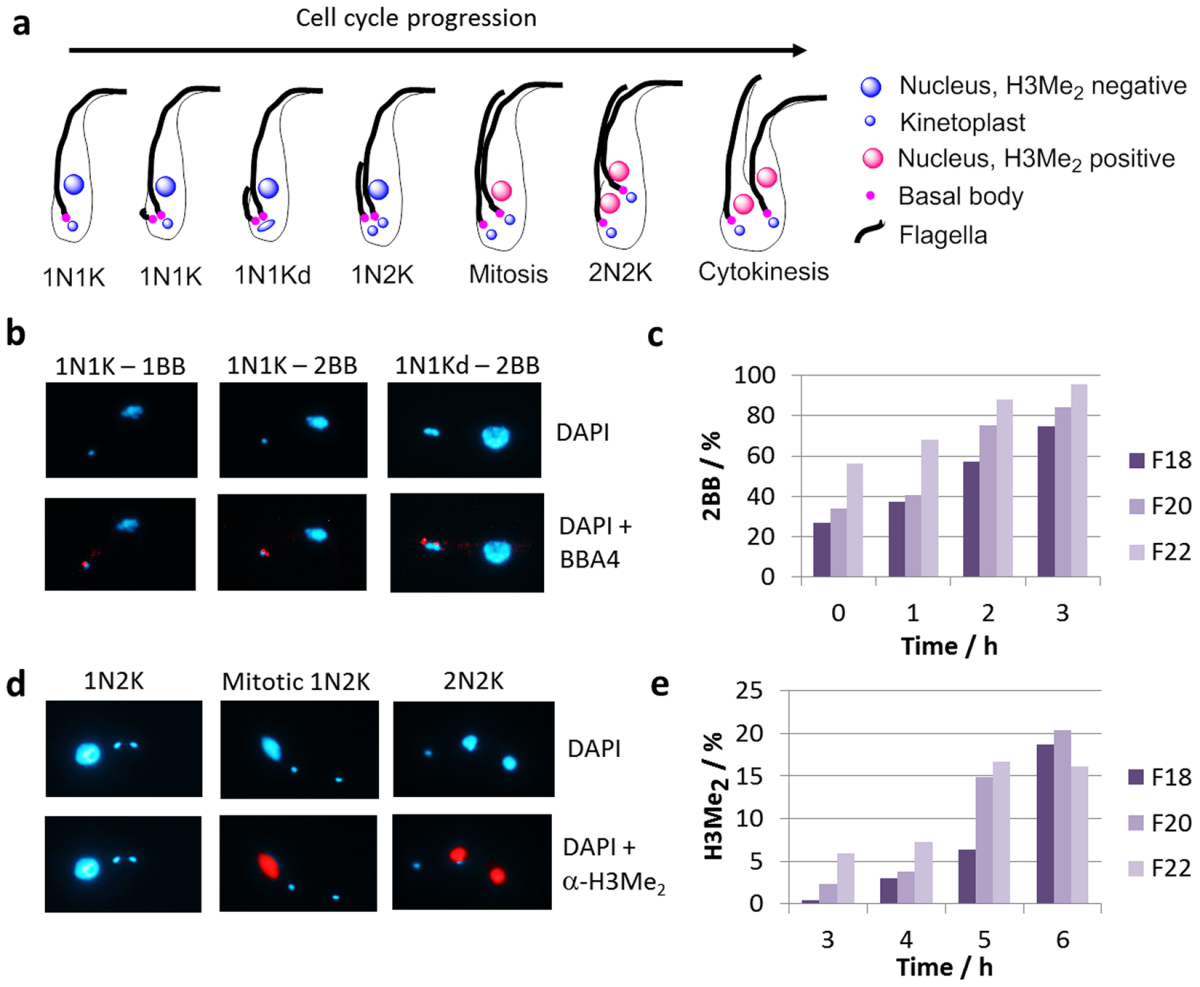
Cytological markers for cell cycle progression in *T. brucei*. (**a**) Schematic of events occurring as cells progress through the cell cycle. (**b**) Immunofluorescence microscopy of Pcf with BBA4 to detect basal bodies. (**c**) Observation of basal bodies in early eluting Pcf fractions. (**d**) Immunofluorescence microscopy of Pcf with α-H3Me_2_ to detect mitotic and post-mitotic cells. (**e**) Observation of α-H3Me_2_ positive cells in early eluting Pcf fractions. F18 - Early-G1 eluted at 18 ml/min, F20 - Mid-G1 cells eluted at 20 ml/min, F22 - Late-G1 cells eluted at 22 ml/min.

Further confirmation that the fractions represented distinct G1-sub populations was sought by using established cytological markers for the beginning and end of the cell cycle (Fig. 4a). The earliest cytological marker available for monitoring cell cycle progression in *T. brucei* is maturation and duplication of the flagellar basal body - probasal body pair, which must occur before kinetoplast duplication and division^24^. Staining with the BBA4 antibody^25^, which recognises both the mature and pro basal bodies (BB), will thus identify early kinetoplast S phase cells by proxy of the appearance of a second mature basal body-probasal body pair. Upon further maturation, this new BB will move around the old BB - flagellum complex and nucleate a new flagellum^26^, a stage which is also marked by elongation of the kinetoplast which can then readily be identified as bilobed (1N1Kd) and about to separate by DAPI staining. Differences in the proportion of 1N1K cells with two mature BBs were already apparent in the starting populations and persisted throughout the following three hours post elutriation (Fig. 4b). Thus, over 60% of 1N1K cells of the 22 ml/min fraction possessed two mature BBs after 1 hour in culture, while it took 2 and 3 hours for the 20 ml/min and the 18 ml/min fractions to reach this point, respectively (Fig. 4c). Overall, there was a steady increase in 1N1K cells with two mature BBs in every fraction; their percentage was always highest in the 22 ml/min fraction, followed by the 20 and 18 ml/min fractions.

Dimethylation of histone H3 (H3Me_2_) is a marker of late G2/mitosis in *T. brucei* ^27^, and it has been shown that about 20% of 1N2K cells and about 90% of 2N2K cells in an asynchronous population are H3Me_2_ positive^28^. To investigate whether the fractions were still temporally separated at later time points post elutriation, we stained cells harvested at 3–6 h post elutriation with the anti- H3Me_2_ antibody (Fig. 4d). A steady increase of the H3Me_2_ mark was observed that was always present in higher percentages in the 20 ml/min fraction when compared with the 18 ml/min fraction, with both of them reaching a maximum of around 20% at 6 hours post elutriation (Fig. 4e). Since the 22 ml/min fraction was more mixed to begin with this fraction reached a slightly lower maximum that plateaued at 17% 5–6 hours post-elutriation. These maximums correlated with the time when most cells in populations were undergoing cytokinesis, as suggested by flow cytometry and DAPI staining (Fig. 3, Supplementary Fig. 4).

### Temporal modelling of the Pcf cell cycle

**Figure 5:**
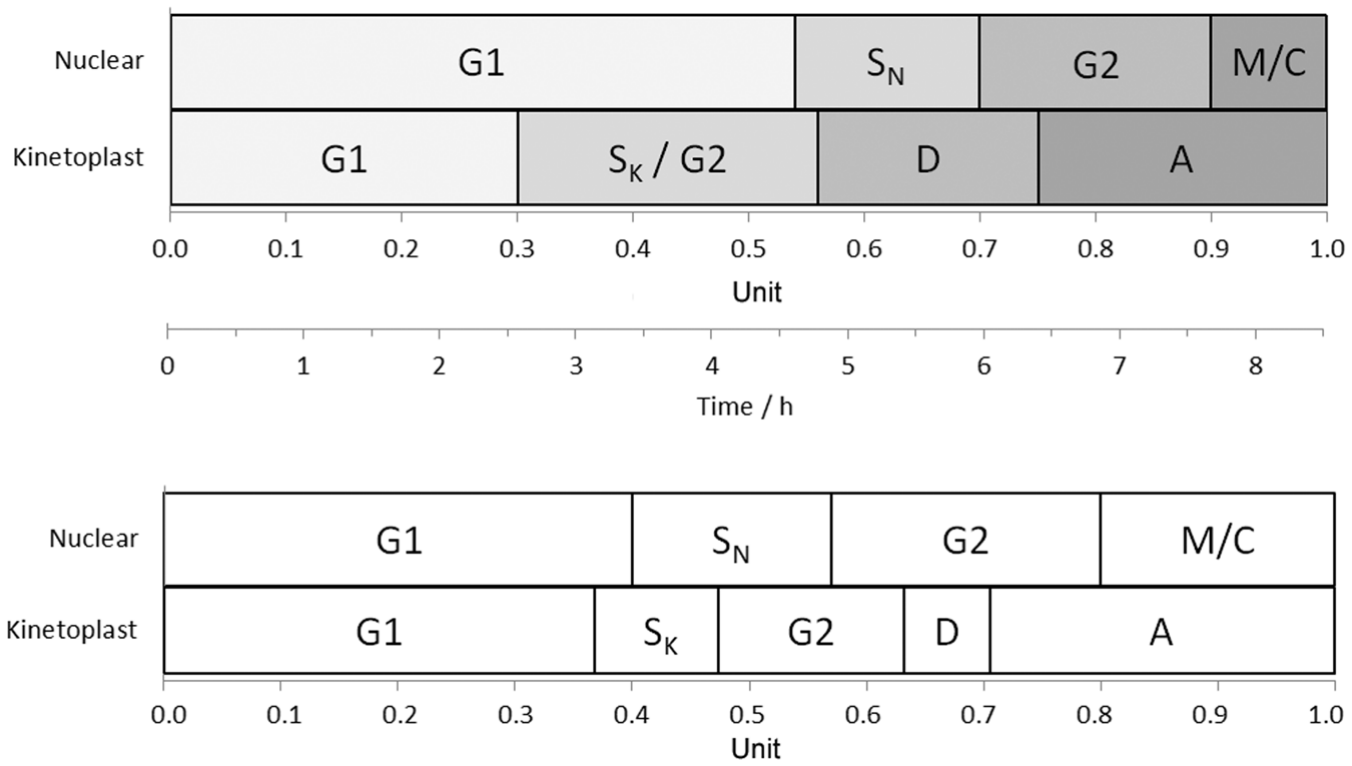
Model of the *T. brucei* cell cycle. (**a**) Model of the relative timing and duration of the nuclear and kinetoplast cell cycle based on temporal observations of early-, mid- and late-G1 Pcf fractions. (**b**) Model proposed by Woodward & Gull based on BrdU labelling of asynchronous cultures, adapted from^29^. The cell cycle phases are as follows: G1 –gap 1, S_N_ – nuclear synthesis, S_K_ – kinetoplast synthesis, G2 - gap 2, M - mitosis, C - cytokinesis, D – kinetoplast division and A – after division.

**Figure 6:**
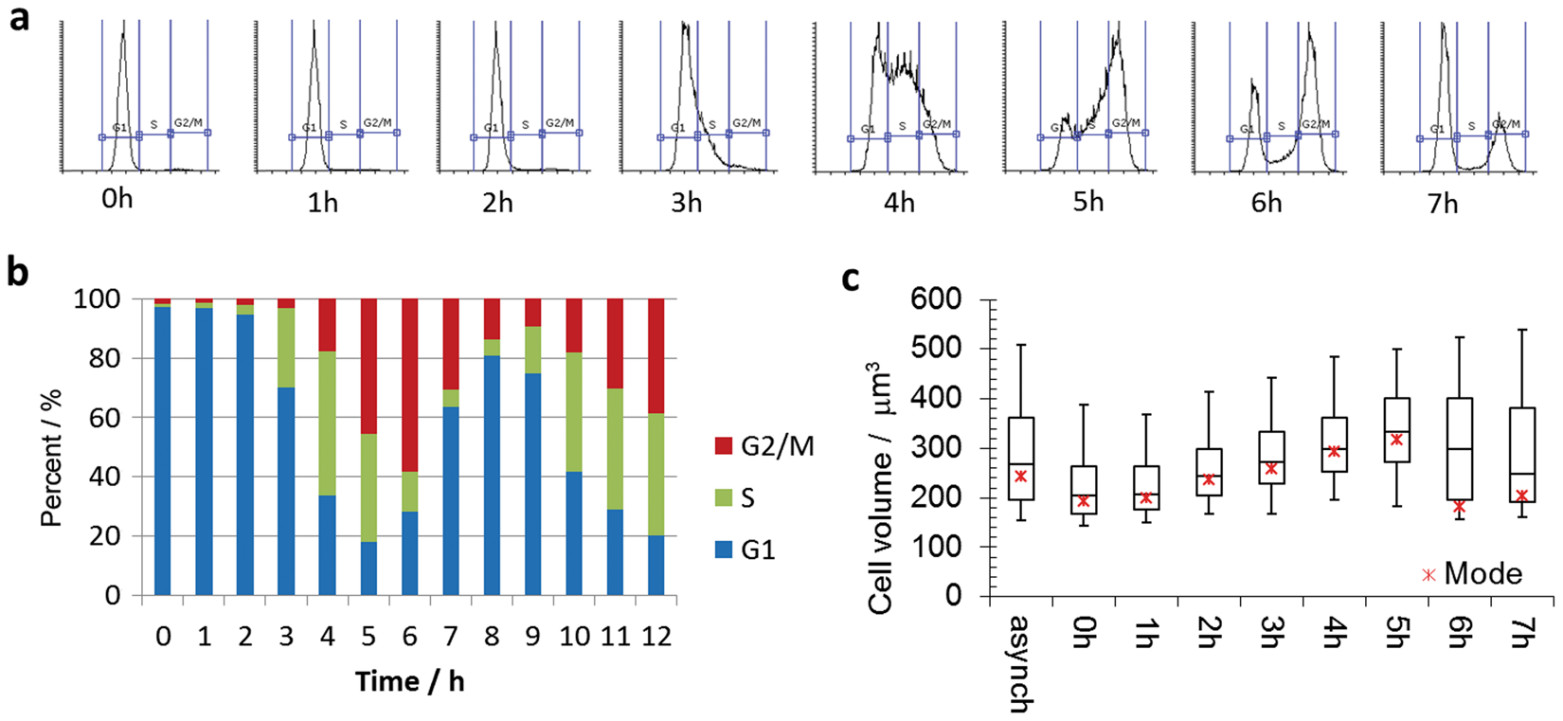
Time-course of early-G1 synchronised Bsf *T. brucei*. Cells samples were withdrawn hourly, stained with PI and analysed by flow cytometry (n = 50,000). (**a**) Flow cytometry trace of first cell cycle post-elutriation. (**b**) Bar chart of flow cytometry data post-elutriation. (**c**) Box plot of cell dimensions determined by CASY model TT (n > 1,500). Boxes represent the median and central quartiles, whiskers represent 9 and 91% of data, and * the mode.

Our observations of the progression of the early-, mid- and late-G1 fractions through the cell cycle were used to construct a model of the relative timing and duration of the nuclear and kinetoplast cell cycle using the nomenclature proposed by Woodward & Gull^29^ (Fig. 5a). We modelled the starting population of cells in each fraction as a latent beta distribution over the cell cycle period, scaled to the unit interval, and assumed that cells all move through the stages of the cell cycle at the same constant rate, so that only the position of the distribution is shifted. The parameters of the beta distribution, as well as the boundaries of the cell cycle stages, were then inferred from the data under a hierarchical Bayesian model via the STAN software package^30^. We used the observation of the maturation of the basal body - probasal body pair to place a lower bound on the kinetoplast S phase, but did not have any data to enable us to distinguish the kinetoplast S phase from G2 phase. Our estimate of the cell cycle phases differs significantly from the those proposed by Woodward and Gull (Fig. 6b) based on analysis of bromo-deoxyuridine (BrdU) labelled asynchronous cells^29^. In particular, we predict that nuclear S phase occurs concurrently with division of the kinetoplast D, consistent with our observations, whereas the Woodward and Gull model predicts that kinetoplast division D occurs during early G2 phase. Notably, our observations that nuclear S phase occurs concurrently with kinetoplast division D are in agreement with the observations made by Siegel *et al*.^13^ and Kabani *et al*.^20^, supporting the validity of our model.

### Elutriation of Bloodstream form cells

Next, we investigated whether counter flow centrifugal elutriation could be used to produce tightly synchronised cell populations of bloodstream form (Bsf) *T. brucei* cells. Due to their smaller size, Bsf cells were loaded into the elutriation chamber at a lower initial flow rate than used with Pcf cells. Incremental increase in the flow rate was use to elute fractions, which were then analysed by flow cytometry and microscopy of DAPI stained slides (Supplementary Figure S5). The early eluting fractions (13, 15 and 17 ml/min) contained highly enriched G1 population (>95%), whereas later fractions were more mixed.

Cells in the early-eluting G1 fractions proliferated synchronously when placed back into culture immediately post-elutriation, without any appreciable lag (Fig. 6). Flow cytometry revealed that a small percentage of cells (<10%) appear to be arrested in G1 and do not move through the cell cycle with the rest of the population; this effect was exacerbated when the cell density of the starting population exceeded 1 × 10^6^ cells/ml, or when cells spent a greater amount of time in the elutriator. For the 15 ml/min G1-fraction, the maximum number of cells in S phase (49%) was reached after 4 hours, and the maximum of number of cells in G2/M phase cells (58%) was reached after 5 h (Fig. 6), representing an enrichment over asynchronous cells of 4.3-fold and 6.3-fold respectively. The cell division time for logarithmically dividing Bsf cells in culture is 7 hours, and after 7 hours 63% of cells had re-entered G1 phase with 31% still in G2/M. Synchronisation was still discernible in the second cell cycle (Fig. 6b), but the maintenance of synchrony into the third cell cycle was not reproducible.

**Figure 7:**
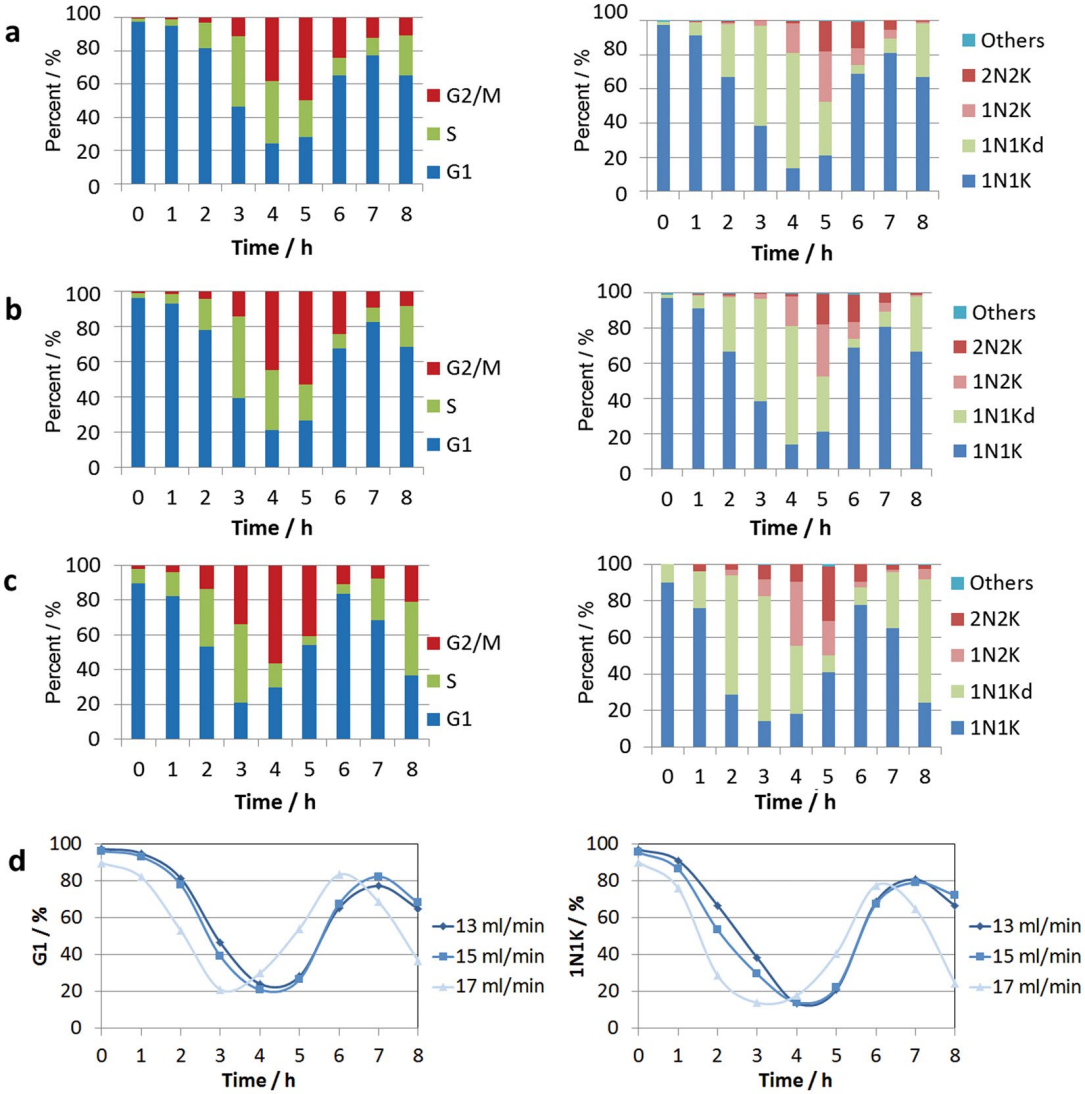
Flow cytometry time-course of G1 synchronised Bsf *T. brucei* fractions. (**a**) Early-G1 cells (97% G1) eluted at 13 ml/min, (**b**) Early-G1 cells (96% G1) eluted at 15 ml/min, (**c**) Late-G1 cells (96% G1) eluted at 17 ml/min. (**d**) Direct comparison of the proportion of G1 (left) and 1N1K cells (right) over time for fractions eluted at 13, 15 and 17 ml/min. Left panel - Flow cytometry of PI stained cells at hourly intervals post elutriation (n = 50,000 per time point). Right panel – Microscopy of DAPI stained cells (n > 200 per time point).

The average cell size for the initial G1 fraction was significantly smaller (p-value 5 × 10^−89^) and more narrowly distributed than the original asynchronous population (Fig. 6c, Supplemental Table S2), and increased as cells progressed through the cell cycle until cytokinesis (Supplemental Fig S3), when the distribution broadened. However, in contrast to the Pcf cells, the average cell size showed little increase in the initial phase of the cell cycle. In agreement with this observation, the progression through the cell cycle of the two earliest eluting G1 fractions (13 & 15 ml/min) were only marginally different, but that of the third G1 fraction (17 ml/min) was distinct (Fig. 7, Supplementary Fig. S6), suggesting that the elutriator is unable to resolve early-G1 from mid-G1 Bsf cells.

## Discussion

A reliable method for cell cycle synchronisation is an invaluable tool to study cell cycle regulation, but the quality of synchronised cells populations produced cannot be easily scored. Cooper has suggested a range of criteria that may be used to judge the quality of synchronisation achieved including: uniform DNA content and narrow size distribution of the initial population, uniform cell number until synchronous division, maintenance of cell division time, and synchronicity that is maintained into successive cell cycles^21,22^. Here, we have demonstrated the centrifugal counter-flow elutriation is able to isolate synchronised population of Bsf and Pcf *T. brucei* that for the first time fulfil all of these criteria.

Cell synchronisation by centrifugal elutriation is a well-established technique in yeast, where it has proved enormously useful in studying cell cycle regulation, and the synchronicity we have achieved with *T. brucei* cells is of comparable quality. The initial enrichment of *T. brucei* cells (96–97% early-G1) is equivalent with that obtained with *S. cerevisiae* (97% G1)^31,32^, where cell cycle enrichment also results in a reduction in the average cell size and the cell size distribution of the population^32^. The synchronicity of Bsf *T. brucei* cells is lost more rapidly than Pcf cells, where synchronicity is still discernible in the third cell cycle, whereas with *S. cerevisiae* synchronicity is maintained into the third cell cycle^33^, although many studies do not look beyond the first cell cycle. However, the ability to produce isolated distinct populations of Pcf cells in early, mid and late G1 phases has, to our knowledge, no precedent in yeast and may prove particularly informative of events occurring early in the cell cycle.

The quality of G1 phase cells obtained in Pcf elutriations (>95%) is superior to any other published method, and good enrichment for subsequent cell cycle phases (up to ~70%) can be obtained by post elutriation culture of the eluted cells. Eluted Pcf cells proliferate synchronously, with synchronicity maintained even during the third cell cycle post elutriation, sufficiently long that RNAi studies of synchronised cell populations may be achievable for proteins with a turnover of <24 h. Moreover, elutriation allows a clear separation of Pcf cells into early, mid and late G1 phase cells from the same culture, allowing parallel analysis of distinct cell populations that cannot be distinguished by PI-stained flow cytometry alone. In addition, it is possible to increase the recovery of cells at a given point in the cell cycle by culturing these fractions for different lengths of time post-elutriation.

We have used our Pcf data to construct the first model of the relative timing and duration of the *T. brucei* nuclear and kinetoplast cell cycle based on temporal observations of synchronised cell populations. Our cell cycle model is a good fit for our observations and those of others^13^, despite the disagreement with the widely used model proposed by Woodward and Gull^29^. Our use of temporal observations precludes the use of the Williams equation^34^ to mathematically correct for the over-observation of G1 phase cells in asynchronous populations. Interestingly, this leads to an increased estimate of the length of G1 in our model compared to those employing the Williams equation^29,35^. To avoid perturbing our cells we have not used labelling strategies to directly observe kinetoplast DNA replication, and thus cannot distinguish the S and G2 phase of the kinetoplast. A recent meticulous study by da Silva *et al*. into the incorporation of BrdU into trypanosomatids concluded that standard detection conditions would significantly underestimate the detection of DNA synthesis^35^. As this effect would be compounded by the small size of the kinetoplast, it may help to explain the discrepancy in the timing of kinetoplast synthesis between the two models.

Bsf cells have once more proven to be more difficult to synchronise, but our technique still allows the isolation of very pure G1 phase cells (>95%) and enrichment of other cell cycle phases (up to ~60%) which is superior to all other available methods. Hydroxyurea-mediated synchronisation will not sufficiently enrich for G1 phase cells, since the proliferating population appears to lose synchronicity during the first cell division following S phase arrest and release^36^. Our Bsf cells are not only viable post elutriation but also proliferate readily when put back into culture, which is not the case with G1 populations obtained by Vibrant Dye cell sorting methods^20^. The synchronicity of the Bsf cells is not as tight as for the Pcf, with synchronicity not maintained during the third cell cycle post elutriation. The smaller increase in cell size at the start of the cell cycle, increased motility and more rapid cell division are the likely factors that prevent more efficient synchronisation. For optimal results, great care needs to be taken about the quality and starting density of the Bsf culture, and the time spent in the elutriator must be kept to a minimum. Despite these caveats, elutriation is a superior method for enrichment of Bsf cells in different cell cycle stages. With only a small percentage (<10%) of cells apparently arrested in G1/G0, the great majority will re-enter a proliferative cell cycle post elutriation. The observation that the proportion of apparently G1/G0 arrested cells increases when the initial cell density is >1 × 10^6^ suggests that even monomorphic Bsf cell lines are capable of modulating their cell division in response to cell density, and warrants further investigation.

In conclusion, we have shown centrifugal elutriation to be a rapid and efficient method for the selection of cell-cycle synchronised populations of both Pcf and Bsf *T. brucei* cells without the necessity for a double-cut elutriation procedure. The procedure is readily scaled, with up to 3 × 10^9^ cells separated in under an hour, and is compatible with metabolic labelling techniques such as SILAC^37^.

## Methods

### Cell culture

Procyclic form (Pcf) *Trypanosoma brucei* Lister 427 cells were grown at 28 °C without CO_2_ in SDM-79, and bloodstream form (Bsf) *Trypanosoma brucei* Lister 427 MITat 1.2 single marker cells^38^ were grown at 37 °C with 5% CO_2_ in HMI-11T^39^ containing 2.5 μg/ml G418.

### Centrifugal counter-flow elutriation

Elutriation was performed in an Avanti J-26S XP centrifuge equipped with a JE 5.0 rotor with a standard 4 mL elutriation chamber (Beckman Coulter) connected to a MasterFlex peristaltic pump (Cole-Palmer) with an in-line pressure gauge and bubble trap prior to the chamber (Fig. 1). Prior to use, the Pcf elutriation buffer (1 × PBS with 25% SDM79) was filter-sterilised through a 0.2 μM filter and degassed in an ultrasonic bath for 15 min, and pre-warmed to 28 °C. Logarithmic phase Pcf cells (5 × 10^6^–2 × 10^7^/ml) were harvested by centrifugation at 1,000 × g for 10 min at room temperature (RT) and resuspended in 20 ml Pcf elutriation buffer. A maximum number of 3 × 10^9^ Pcf were used for each elutriation. The centrifuge was set at a constant 5,000 rpm without cooling and the elutriation chamber was equilibrated with Pcf elutriation buffer at 10 ml/min. The peristaltic pump was used to load the Pcf cells at 10 ml/min until a stable boundary became visible in the elutriation chamber viewing port. The pump speed was then incrementally increased and 150 ml fractions of eluted cells collected. Eluted cells were counted, harvested by centrifugation at 1,000 × g for 10 min at RT, resuspended in the appropriate amount of SDM-79 and transferred to a 28 °C incubator without CO_2_ for further cultivation. Flow cytometry samples and microscopy slides were prepared at different time points following elutriation as specified in the respective figures.

Elutriation of Bsf cells was performed as above, with the following changes: i. logarithmic phase Bsf cells (<1 × 10^6^/ml) were harvested by centrifugation at 800 × g for 10 min at RT and resuspended in 20 ml of filter-sterilised and degassed Bsf elutriation buffer (1 × PBS with 25% HMI-11T and 10 g/L glucose) pre-warmed to 37 °C, ii. cells were loaded at 8 ml/min, iii. 100 ml fractions were collected and resuspended in HMI-11T before culture at 37 °C with 5% CO_2_.

### Flow cytometry

Approximately 2 × 10^6^ cells were pelleted, washed once with PBS and fixed in 1 ml of 70% methanol in 1 × PBS and stored at 4 °C overnight. Following a PBS wash, samples were incubated with 10 µg/ml Propidium iodide (Sigma) and 9.6 µg/ml of RNAseA (Sigma) at 37 °C for 45 min. Samples were analysed on a FACS Canto II (BD) collecting 50,000 gated events, and data processed in Flowing Software 2.5.1 (http://www.uskonaskel.fi/flowingsoftware/) using doublet discrimination and automatic assignment of cell cycle boundaries according to the Propidium iodide intensity.

### Immunofluorescence and DAPI counts

For basal body staining with BBA4^25^, Pcf cells were washed in 1 × PBS, spread onto glass slides, allowed to settle for 5 min at room temperature (RT), then fixed with 4% paraformaldehyde in 1 × PBS for 10 min at RT. Following storage and permeabilisation in 100% methanol at −20 °C for 20 min, slides were rehydrated in 1 × PBS for 5 min before proceeding with antibody staining.

For staining dimethylated histone H3 with anti-H3Me_2_ antibody^27^, Pcf cells were washed with 1 × PBS, fixed in 2% paraformaldehyde in 1 × PBS for 10 min at 4 °C, spread onto a glass slide and allowed to adhere, and then permeabilised for 10 min in 0.2% NP-40 in 1 × PBS at RT.

Primary antibody (anti-BBA4, at 1:50 dilution; anti-H3Me_2_, at 1:1,000 dilution in 1 × PBS) was added to the slides and incubated for 1 h at room temperature (BBA4) or at 4 °C overnight (H3Me_2_). Following two washes with 1 × PBS, secondary antibody (Rhodamine-coupled rabbit anti-mouse IgM, AlexaFluor596-coupled goat anti-rabbit IgG; both at 1:200 and purchased from Sigma/Molecular Probes) was added, and the slides incubated for 1 h at RT. Following two washes with 1 × PBS, the slides were sealed with Fluoroshield (Sigma) and examined on a Leica DMRXA2 fluorescent microscope. Basal bodies of 1N1K/1N2K cells were counted for a minimum of 200 cells per fraction and time point. H3Me_2_-positive cells were counted and expressed as a percentage of the whole population for at least 170 cells per time point.

For DAPI counts, cells were spread onto glass slides, allowed to air-dry at RT and then fixed and permeabilised in 100% methanol at −20 °C. Slides were removed from methanol, allowed to air-dry at RT and then rehydrated in 1 × PBS. DAPI (0.1 µg/ml, Sigma) was added and the slides examined on a Leica DMRXA2 fluorescent microscope. At least 200 cells were counted per time point for each fraction and scored according to their number of nuclei (N) and kinetoplasts (K) (Supplementary Figure S7), and biological triplicates were performed to enable quantitative analysis.

### Cell Dimensions

Cell dimensions were determined using a CASY Model TT cell counter and analyser (Innovatis) fitted with a 60 μM capillary. Cells were diluted to an appropriate concentration in CASYton to allow analysis of >1,500 cells, and data was exported using CASYWorx 1.21 (OMNI Life Science) for further statistical analysis in excel. The two-tailed student’s T-test was used to calculate the significance of differences in the cell sizes observed.

### Cell cycle model

We model the latent distribution of the starting population of cells in each fraction at the start of the experiment as part of a hierarchical Bayesian model with a beta distribution over the unit cell cycle. The parameters of the beta distribution, as well as the boundaries of the cell cycle stages, were inferred from the data using Hamiltonian Monte Carlo via the STAN software package^30^. Further details of the model are given in the supplementary data.

### Data availability statement

All data generated or analysed during this study are included in this published article and its Supplementary Information files.

## Electronic supplementary material

Supplementary information

Supplementary Table S1

Supplementary Table S2

## References

[1] Vasquez J-J, Hon C-C, Vanselow JT, Schlosser A, Siegel TN (2014). Comparative ribosome profiling reveals extensive translational complexity in different Trypanosoma brucei life cycle stages. Nucleic Acids Res..

[2] Manful T, Fadda A, Clayton C (2011). The role of the 5′−3′ exoribonuclease XRNA in transcriptome-wide mRNA degradation. RNA.

[3] Clayton C (2013). The Regulation of Trypanosome Gene Expression by RNA-Binding Proteins. PLoS Pathog..

[4] Kolev NG, Ramey-Butler K, Cross GAM, Ullu E, Tschudi C (2012). Developmental Progression to Infectivity in Trypanosoma brucei Triggered by an RNA-Binding. Protein. Science (80-.)..

[5] Walrad PB, Capewell P, Fenn K, Matthews KR (2012). The post-transcriptional trans-acting regulator, TbZFP3, co-ordinates transmission-stage enriched mRNAs in Trypanosoma brucei. Nucleic Acids Res..

[6] Wurst M (2012). Expression of the RNA recognition motif protein RBP10 promotes a bloodstream-form transcript pattern in Trypanosoma brucei. Mol. Microbiol..

[7] Hammarton TC (2007). Cell cycle regulation in Trypanosoma brucei. Mol. Biochem. Parasitol..

[8] Li Z (2012). Regulation of the cell division cycle in Trypanosoma brucei. Eukaryot. Cell.

[9] McKean PG (2003). Coordination of cell cycle and cytokinesis in Trypanosoma brucei. Curr. Opin. Microbiol..

[10] Archer, S. K., Inchaustegui, D., Queiroz, R. & Clayton, C. The cell cycle regulated transcriptome of Trypanosoma brucei. *PLoS One* **6** (2011). 10.1371/journal.pone.0018425 PMC3069104 21483801

[11] Zhou, Q., Hu, H. & Li, Z. In *International Review of Cell and Molecular Biology* **308**, 127–166 (2014). 10.1016/B978-0-12-800097-7.00004-X PMC4374570 24411171

[12] Jones NG (2014). Regulators of Trypanosoma brucei Cell Cycle Progression and Differentiation Identified Using a Kinome-Wide RNAi Screen. PLoS Pathog..

[13] Siegel TN, Hekstra DR, Cross GAM (2008). Analysis of the Trypanosoma brucei cell cycle by quantitative DAPI imaging. Mol. Biochem. Parasitol..

[14] Wheeler RJ, Scheumann N, Wickstead B, Gull K, Vaughan S (2013). Cytokinesis in *Trypanosoma brucei* differs between bloodstream and tsetse trypomastigote forms: implications for microtubule-based morphogenesis and mutant analysis. Mol. Microbiol..

[15] Wheeler RJ, Gull K, Gluenz E (2012). Detailed interrogation of trypanosome cell biology via differential organelle staining and automated image analysis. BMC Biol..

[16] Morriswood, B. & Englester, M. Let’s get fISSical: fast in silico synchronization as a new tool for cell division cycle analysis. *Parasitology* 1–14 (2017). 10.1017/S0031182017000038 PMC5964468 28166845

[17] Hughes L, Borrett S, Towers K, Starborg T, Vaughan S (2017). Patterns of organelle ontogeny through a cell cycle revealed by whole-cell reconstructions using 3D electron microscopy. J. Cell Sci..

[18] Gale M, Carter V, Parsons M (1994). Cell cycle-specific induction of an 89 kDa serine/threonine protein kinase activity in Trypanosoma brucei. J. Cell Sci..

[19] Chowdhury AR, Zhao Z, Englund PT (2008). Effect of hydroxyurea on procyclic Trypanosoma brucei: An unconventional mechanism for achieving synchronous growth. Eukaryot. Cell.

[20] Kabani S, Waterfall M, Matthews KR (2010). Cell-cycle synchronisation of bloodstream forms of Trypanosoma brucei using Vybrant DyeCycle Violet-based sorting. Mol. Biochem. Parasitol..

[21] Cooper S (2004). Is whole-culture synchronization biology’s perpetual-motion machine?. Trends Biotechnol..

[22] Cooper S (2003). Rethinking synchronization of mammalian cells for cell cycle analysis. Cell. Mol. Life Sci..

[23] Venet F, Guignant C, Monneret G (2011). Cell Cycle Synchronization. Methods Mol. Biol..

[24] Sherwin, T. & Gull, K. The Cell Division Cycle of Trypanosoma brucei brucei: Timing of Event Markers and Cytoskeletal Modulations. *Philos. Trans. R. Soc. London B Biol. Sci*. **323** (1989). 10.1098/rstb.1989.0037 2568647

[25] Woodward, R., Carden, M. J. & Gull, K. Immunological characterization of cytoskeletal proteins associated with the basal body, axoneme and flagellum attachment zone of Trypanosoma brucei. *Parasitology* 77–85 (1995). 10.1017/s0031182000064623 7609993

[26] Gluenz E, Povelones ML, Englund PT, Gull K (2011). The kinetoplast duplication cycle in Trypanosoma brucei is orchestrated by cytoskeleton-mediated cell morphogenesis. Mol. Cell. Biol..

[27] Janzen CJ, Hake SB, Lowell JE, Cross GAM (2006). Selective Di- or Trimethylation of Histone H3 Lysine 76 by Two DOT1 Homologs Is Important for Cell Cycle Regulation in Trypanosoma brucei. Mol. Cell.

[28] Gassen A (2012). DOT1A-dependent H3K76 methylation is required for replication regulation in Trypanosoma brucei. Nucleic Acids Res..

[29] Woodward R, Gull K (1990). Timing of nuclear and kinetoplast DNA replication and early morphological events in the cell cycle of Trypanosoma brucei. J. Cell Sci..

[30] Carpenter B (2017). *Stan*: A Probabilistic Programming Language. J. Stat. Softw..

[31] Marbouty M, Ermont C, Dujon B, Richard GF, Koszul R (2014). Purification of G1 daughter cells from different Saccharomycetes species through an optimized centrifugal elutriation procedure. Yeast.

[32] Gordon, C. N. & Elliott, S. C. Fractionation of Saccharomyces cerevisiae Fractionation of Saccharomyces cerevisiae Cell Populations by Centrifugal Elutriation. **129**, 97–100 (1977). 10.1128/jb.129.1.97-100.1977 PMC234900 318655

[33] Hagan IM, Grallert A, Simanis V (2016). Cell cycle synchronization of Schizosaccharomyces pombe by centrifugal elutriation of small cells. Cold Spring Harb. Protoc..

[34] Williams, F. M. In *Systems Analysis and Simulation in Ecology* 197–267 (1971).

[35] da Silva, M. S., Muñoz, P. A. M., Armelin, H. A. & Elias, M. C. Differences in the Detection of BrdU/EdU Incorporation Assays Alter the Calculation for G1, S, and G2 Phases of the Cell Cycle in Trypanosomatids. *J. Eukaryot. Microbiol*., 10.1111/jeu.12408 (2017). 10.1111/jeu.12408 28258618

[36] Forsythe GR, McCulloch R, Hammarton TC (2009). Hydroxyurea-induced synchronisation of bloodstream stage Trypanosoma brucei. Mol. Biochem. Parasitol..

[37] Urbaniak MD, Guther MLS, Ferguson MaJ (2012). Comparative SILAC proteomic analysis of Trypanosoma brucei bloodstream and procyclic lifecycle stages. PLoS One.

[38] Wirtz E, Leal S, Ochatt C, Cross GA (1999). A tightly regulated inducible expression system for conditional gene knock-outs and dominant-negative genetics in Trypanosoma brucei. Mol. Biochem. Parasitol..

[39] Urbaniak MD, Martin DMa, Ferguson MAJ (2013). Global quantitative SILAC phosphoproteomics reveals differential phosphorylation is widespread between the procyclic and bloodstream form lifecycle stages of Trypanosoma brucei. J. Proteome Res..

