# Supplementary material for: Cell cycle synchronisation of Trypanosoma brucei by centrifugal counter-flow elutriation reveals the timing of nuclear and kinetoplast DNA replication
Source: Sci Rep. 2017 Dec 14;7:17599. doi: 10.1038/s41598-017-17779-z (PMC5730572; doi:10.1038/s41598-017-17779-z)
Supplement: Supplementary file 1 — Supplementary information [file 41598_2017_17779_MOESM1_ESM.pdf]

Cell cycle synchronisation of *Trypanosoma brucei* by centrifugal counter-flow elutriation reveals the timing of nuclear and kinetoplast DNA replication

Corinna Benz,[1] Frank Dondelinger,[2] Paul G. McKean[1] and Michael D. Urbaniak[1]*

[1] Division of Biomedical and Life Sciences, Faculty of Health and Medicine, Lancaster University, Lancaster LA1 4YG, UK

[2] Lancaster Medical School, Faculty of Health and Medicine, Lancaster University, Lancaster LA1 4YG, UK

* Corresponding author

E-mail: m.urbaniak@lancaster.ac.uk

Supplementary Information

Table of Contents

Supplementary Figures 2

Figure S1. Flow cytometry analysis of Pcf *T. brucei* isolated by centrifugal elutriation. 2

Figure S2. Analysis of Pcf *T. brucei* isolated by centrifugal elutriation. 2

Figure S3. Flow cytometry time-course of early-G1 synchronised Pcf *T. brucei*. 3

Figure S4. Flow cytometry time-course of distinct G1 synchronised Pcf *T. brucei* cells. 3

Figure S5. Flow cytometry analysis of Bsf *T. brucei* isolated by centrifugal elutriation. 4

Figure S6. Flow cytometry time-course of G1 synchronised Bsf T. brucei. 5

Figure S7. Observation of Nuclear and Kinetoplast DNA by DAPI staining. 5

Cell cycle modelling 6

Figure S8. Estimates of the posterior boundaries for the Pcf *T. brucei* cell cycle. 8

Figure S9. Comparison of the inferred model and Pcf flow cytometry data. 8

Figure S10. Comparison of the inferred model and DAPI microscopy data. 9

Figure S11. Comparison of the inferred model and basal body data. 9

Supplementary Figures

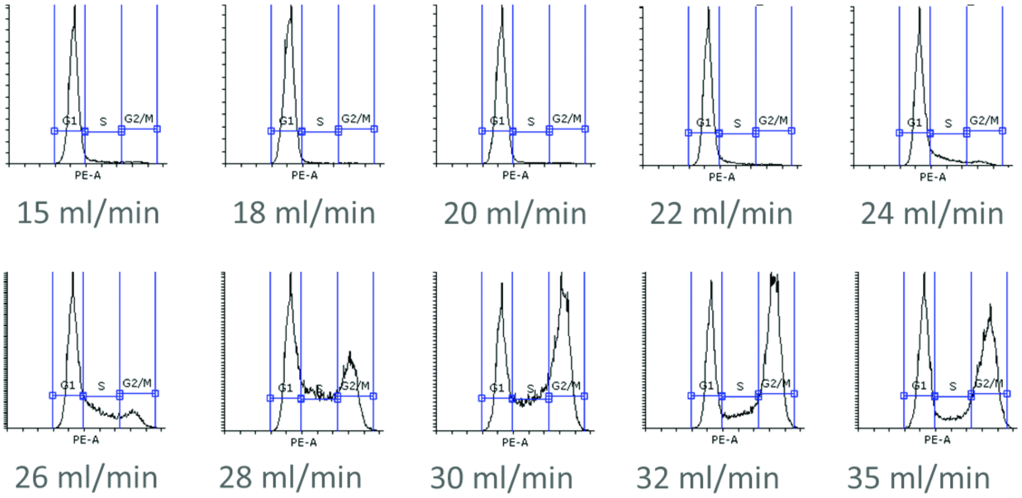

Figure S1. Flow cytometry analysis of Pcf *T. brucei* isolated by centrifugal elutriation. Cells were eluted from the centrifuge with increasing flow rates, stained with PI and analysed by flow cytometry (n = 50,000).

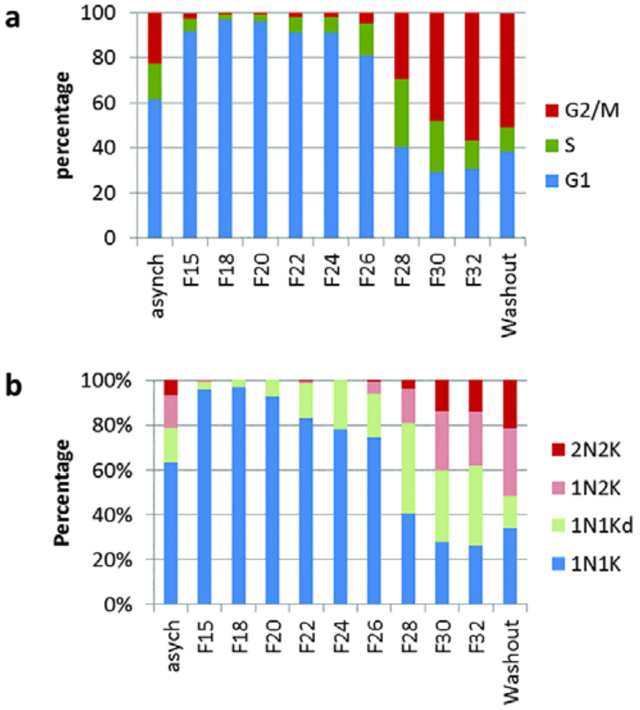

Figure S2. Analysis of Pcf *T. brucei* isolated by centrifugal elutriation. a. Cells were eluted from the centrifuge with increasing flow rates, stained with PI and analysed by flow cytometry (n = 50,000). **b.** Cells were stained with DAPI and the number of nuclei and kinetoplast per cell analysed by microscopy (n > 200).

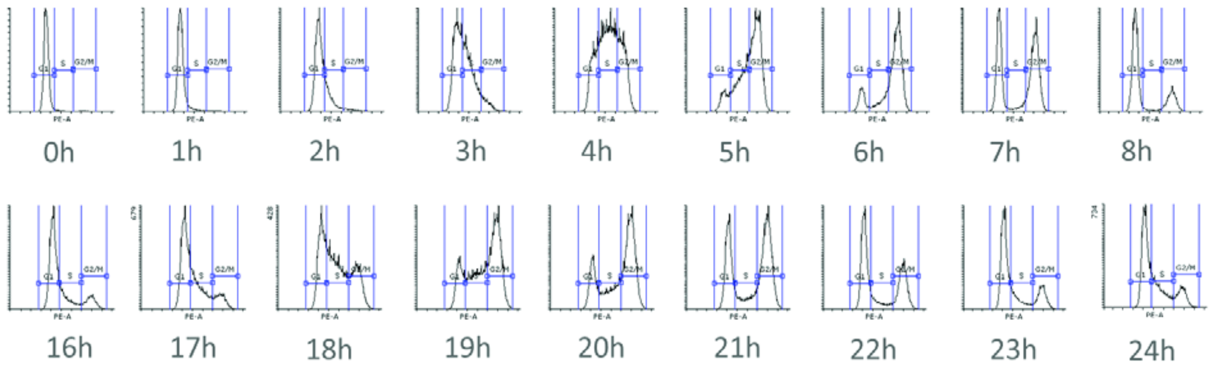

Figure S3. Flow cytometry time-course of early-G1 synchronised Pcf *T. brucei*. Early-G1 cells (97% G1) eluted at 18 ml/min were placed into culture and sample withdrawn hourly, PI stained, and analysed by flow cytometry (n = 100,000).

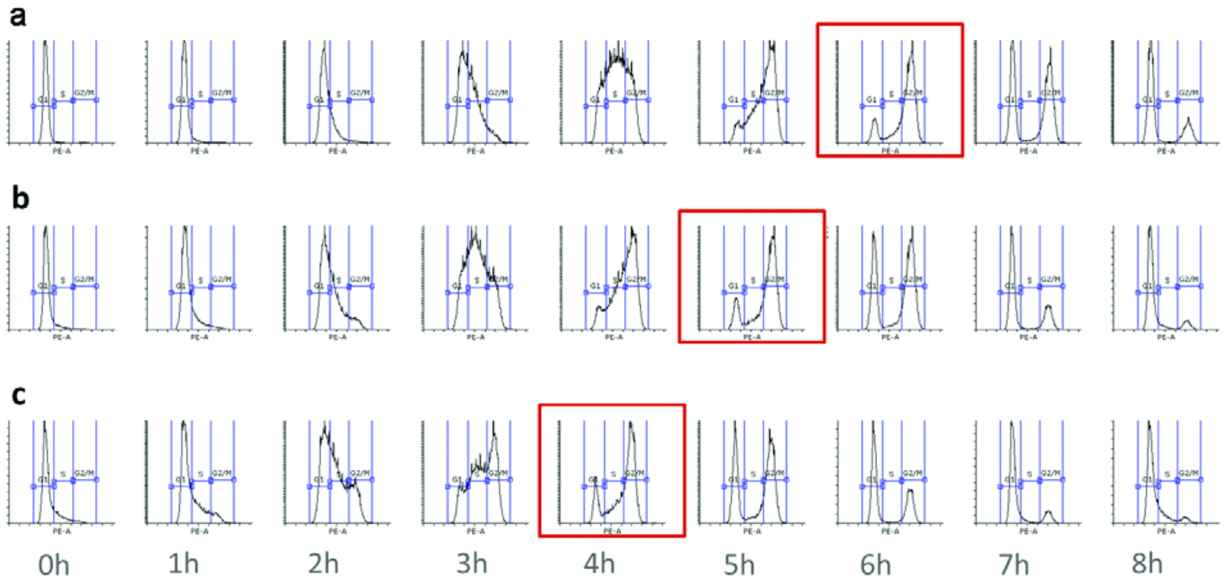

Figure S4. Flow cytometry time-course of distinct G1 synchronised Pcf *T. brucei* cells. Flow cytometry of PI stained cells at hourly intervals post elutriation, details as Fig S1. **a.** Early-G1 cells (97% G1) eluted at 18 ml/min, **b.** Mid-G1 cells (92% G1) eluted at 20 ml/min, **c.** Late-G1 cells (82% G1) eluted at 22 ml/min. Red boxes highlight a similar highly enriched G2/M state that is reached at different time post-elutriation for each sample.

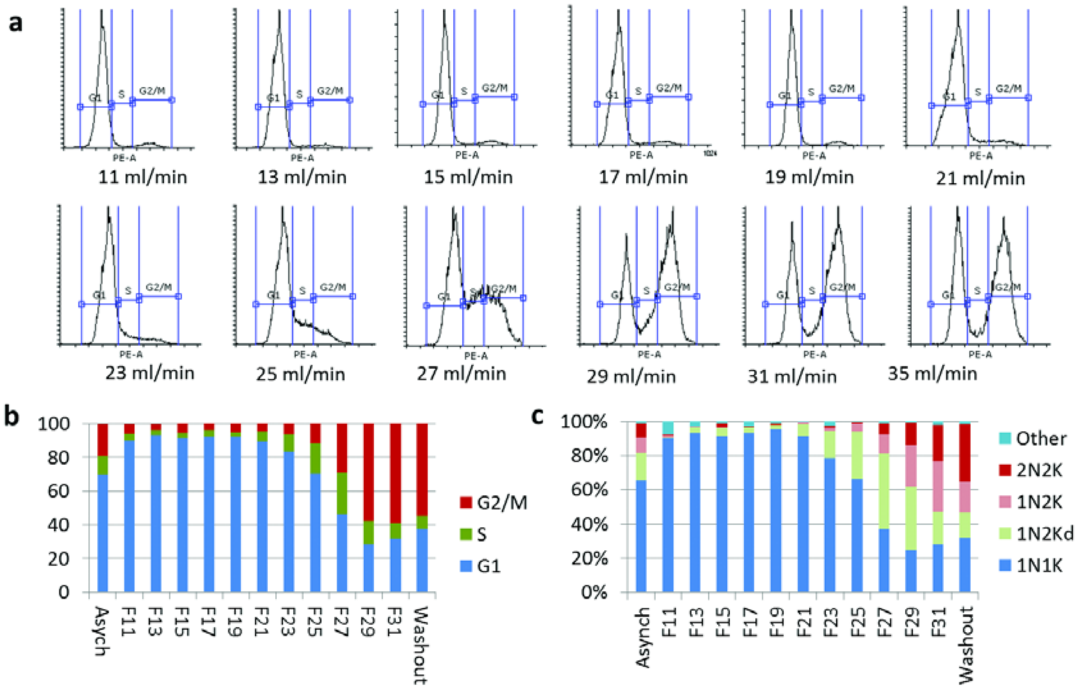

Figure S5. Flow cytometry analysis of Bsf *T. brucei* isolated by centrifugal elutriation. **a.** Cells were eluted from the centrifuge with increases in the flow rates, stained with PI and analysed by flow cytometry (n = 50,000). **b**. Bar chart of flow cytometry data. **c**. Cells were stained with DAPI and the number of nuclei and kinetoplast per cell analysed by microscopy (n > 200).

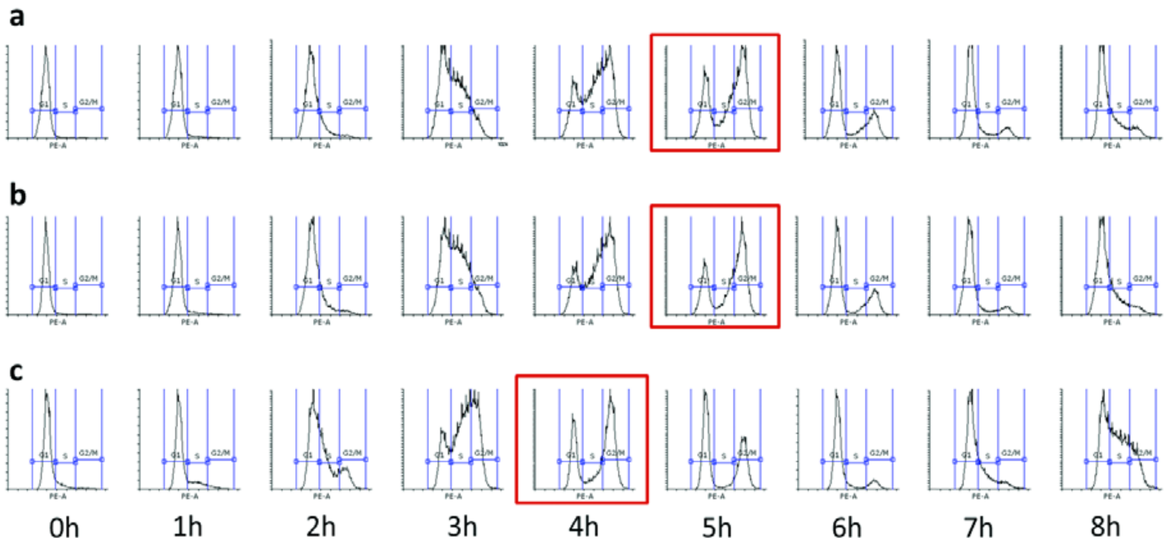

Figure S6. Flow cytometry time-course of G1 synchronised Bsf T. brucei. a. Early-G1 cells (97% G1) eluted at 13 ml/min, **b.** Early-G1 cells (96% G1) eluted at 15 ml/min, **c.** Late-G1 cells (97% G1) eluted at 17 ml/min. Flow cytometry of PI stained cells at hourly intervals post elutriation. Red box highlights a similar profile that is reached at different time post-elutriation for the early- and late-G1 cells.

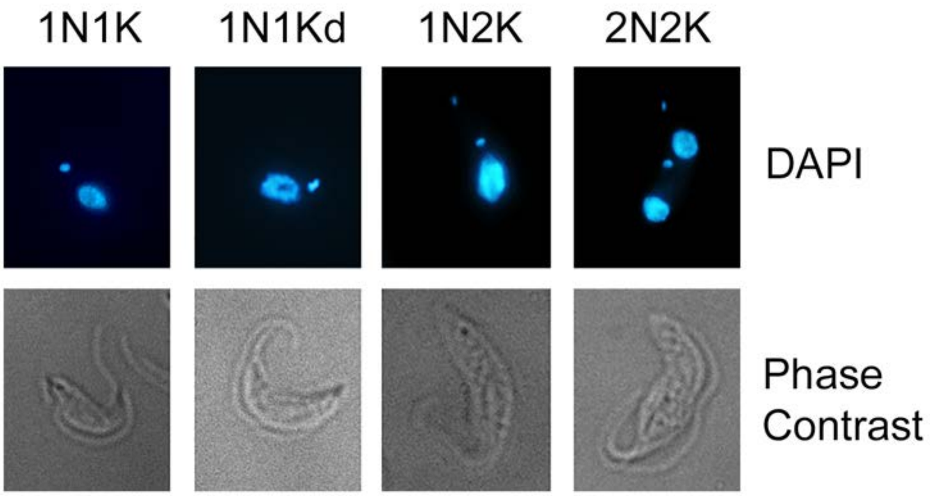

Figure S7. Observation of Nuclear and Kinetoplast DNA by DAPI staining. Examples of the different classifications of cells based on their Nuclear (N) and Kinetoplast (K) count.

Cell cycle modelling

We model the distribution of the starting population of cells in each fraction at the start of the experiment over the unit cell cycle with a latent beta distribution with shape and scale parameters α and β. We assume that the cells all move through the stages of the cell cycle at the same constant rate, and so the position of the distribution will shift. Hence, we define $\mu = a_0 + a_1 t$, where t denotes the time of measurement. The beta distribution can be reparameterised in terms of α and a_0, with $\beta = \frac{\alpha}{a_0 - \alpha}$.[1]

Conceptually, we are interested in inferring the boundaries of major cell cycle stages from the data. Let Θ be the vector encoding the $K+1$ boundaries of the K observed cell cycle stages (whether overall cell cycle, kinetoplast, or basal body state). For example, $\Theta_{cc} = \{0, \theta_{G1}, \theta_S, \theta_{G2/M}, 1\}$ for the overall cell cycle. Let Y be the vector of observed proportions of cells in each stage.

First, let us assume that we are only looking at a single cell cycle. We can model the data using the cumulative distribution function $F(u \parallel \alpha, \beta)$ of the beta distribution. Let $F(u, v, t)$ denote the probability mass between points u and v under the shift at time t, i.e. $F(u, v) = F(v - a_1 t) - F(u - a_1 t)$, where we have omitted α and β for simplicity. Note that we assume the probability mass to be zero outside the unit interval, so $F(u, v, t) = F(v - a_1 t) - F(0)$ if $u - a_1 t < 0$ and $F(u, v) = F(1) - F(u - a_1 t)$ if $v - a_1 t > 1$. Then allowing for a small amount of Gaussian observational noise with variance σ^2, our model for cell cycle stage k is:

$$Y_k(t) \sim N(F(\Theta_k, \Theta_{k+1}, t), \sigma^2).$$

In order to model the doubling of cells at the end of each cell cycle, we must introduce a discontinuity into the model. As the duration of the experiment only allows for two cell cycles, we can determine the proportion of cells in each cycle under our model: $p_1(t) = F(0,1,t)$ and $p_2(t) = F(1,2,t)$, where we have doubled the unit cell cycle in the obvious way. Then we can define a normalized version of $F(u, v, t)$:

$$F_{norm}(u, v, t) = \frac{F(u, v, t) + 2F(u+1, v+1, t)}{p_1(t) + 2 * p_2(t)}$$

and the model becomes:

1 Note that the beta distribution is only defined on the unit interval, but we need cells to be able to proceed to the next cell cycle as time progresses. We solve this by shifting the reference frame.

$$Y_k(t) \sim N(F_{norm}(\Theta_k, \Theta_{k+1}, t), \sigma^2).$$

If we define $F(\Theta, t \parallel \alpha, a_0, a_1) = \{F_{norm}(\Theta_1, \Theta_2, t), \dots, F_{norm}(\Theta_K, \Theta_{K+1}, t)$, then we can redefine the equation above as $Y(t) \sim N(F(\Theta, t \parallel \alpha, a_0, a_1), \sigma^2)$. We define priors on the inferred parameters α, a_0, a_1 and Θ, leading to a Bayesian hierarchical model:

$$\begin{gathered}
Y_{nuclear}(t) \sim N\big(F(\Theta_{nuclear}, t \parallel \alpha, a_0, a_1), \sigma^2_{nuclear}\big) \\
Y_{kinetoplast}(t) \sim N\big(F\big(\Theta_{kinetoplast}, t \parallel \alpha, a_0, a_1\big), \sigma^2_{kinetoplast}\big) \\
Y_{basal}(t) \sim N\big(F(\Theta_{basal}, t \parallel \alpha, a_0, a_1), \sigma^2_{basal}\big) \\
\Theta_{nuclear} \sim N\big(B_{nuclear}, \sigma^2_{bound}\big) \\
\Theta_{kinetoplast} \sim N\big(B_{kinetoplast}, \sigma^2_{bound}\big) \\
\Theta_{basal} \sim N\big(B_{basal}, \sigma^2_{bound}\big) \\
\alpha \sim Gamma(1,1) \\
a_0 \sim N(0.3, 0.05) \\
a_1 \sim N(0.02, 0.01) \\
\sigma_{nuclear}, \sigma_{kinetoplast}, \sigma_{basal} \sim Exponential(10)
\end{gathered}$$

where B encodes our prior beliefs about the location of the boundaries, with $B_{nuclear} = \{0.6, 0.75\}$, $B_{kinetoplast} = \{0.1, 0.3, 0.6\}$ and $B_{basal} = \{0.5\}$. Note that the start and end of the cycle are fixed, so do not require priors. We set $\sigma_{bound} = 0.1$, though in practice the posterior estimates of the bounds will be tightly constrained by the data.

We infer the parameters using Hamiltonian Monte Carlo via the STAN software package[1]. The model converges after 5000 iterations, and produces the estimates of the posterior boundaries shown in Figure S8. The fit of the data for each of the three fractions to the inferred model is shown for the flow cytometry data (Fig. S9), the DAPI stained microscopy data (Fig S10) and the basal body count (Fig. S11).

References:

1. Carpenter, B. *et al.* (2017) *Stan* : A Probabilistic Programming Language. *J. Stat. Softw.* **76,** 1–32

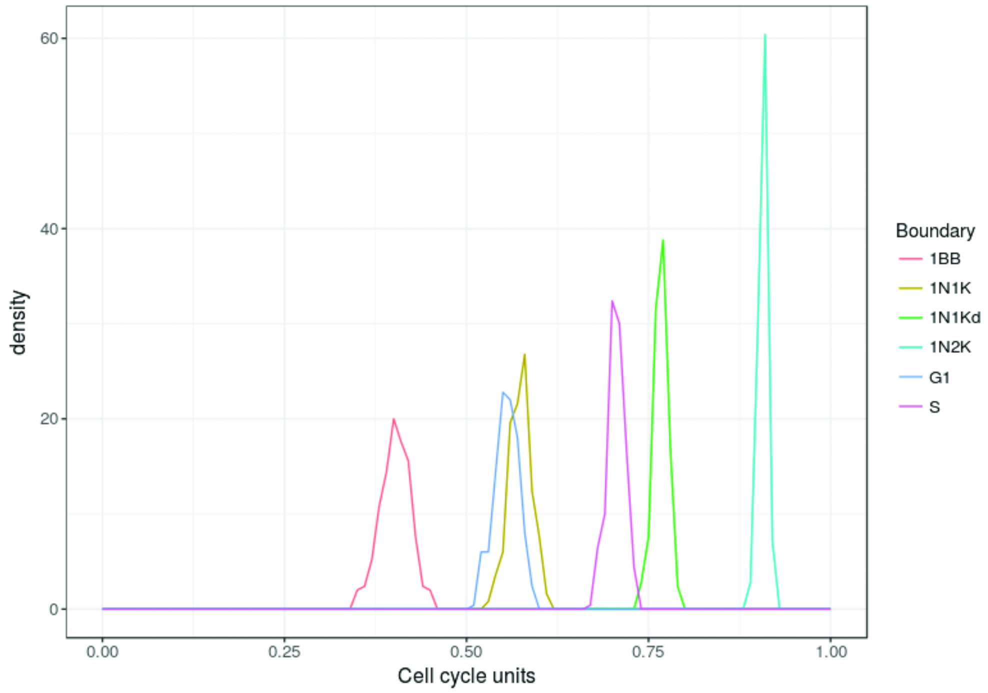

Figure S8. Estimates of the posterior boundaries for the Pcf *T. brucei* cell cycle.

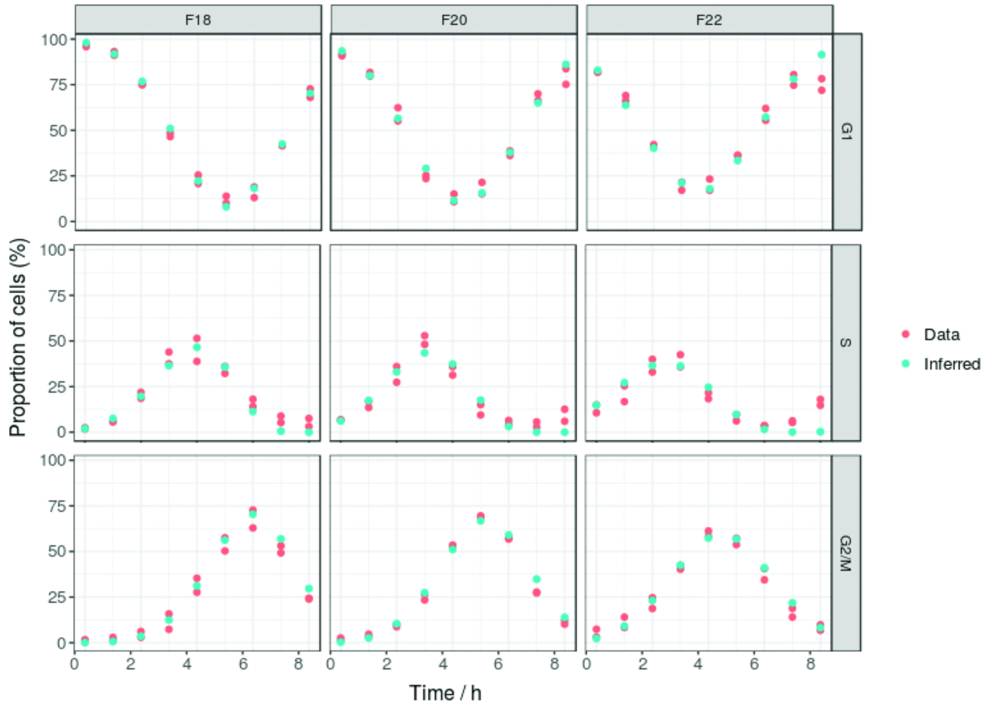

Figure S9. Comparison of the inferred model and Pcf flow cytometry data. F18 – 18 ml/min fraction, F20 – 20 ml/min fraction, F22 22 – ml/min fraction. Two data sets used.

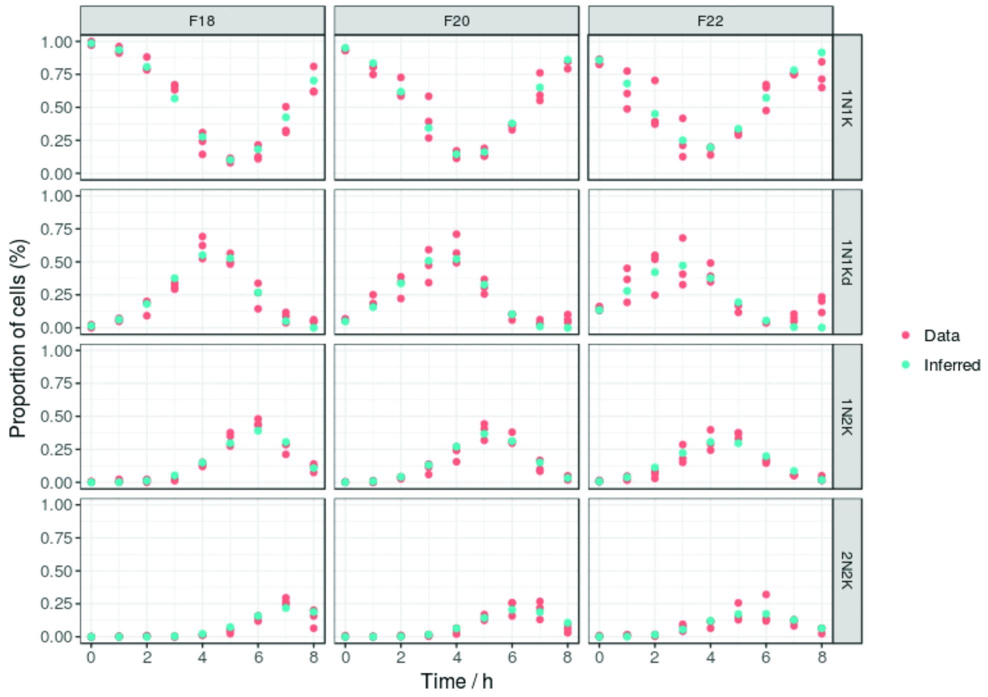

Figure S10. Comparison of the inferred model and DAPI microscopy data. F18 – 18 ml/min fraction, F20 – 20 ml/min fraction, F22 22 – ml/min fraction. Three data sets used.

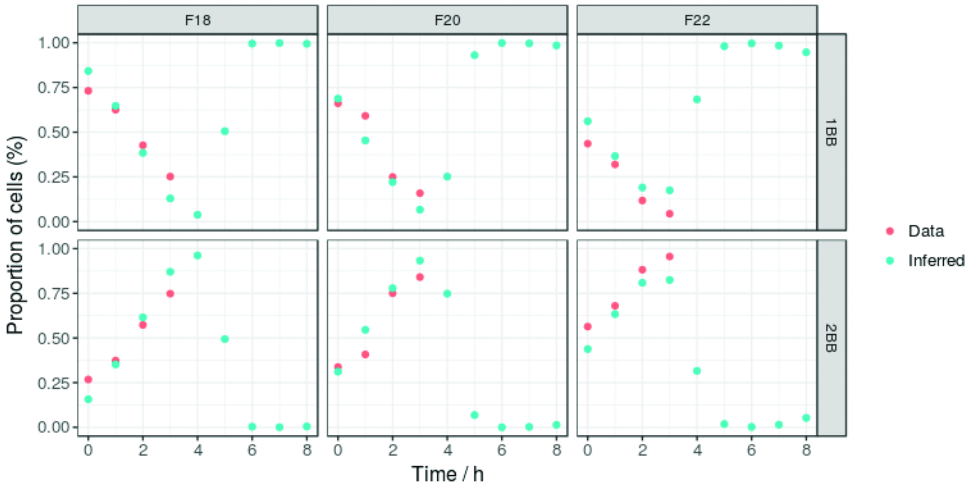

Figure S11. Comparison of the inferred model and basal body data. F18 – 18 ml/min fraction, F20 – 20 ml/min fraction, F22 22 – ml/min fraction. Single data set used.
